# Bioglass-Reinforced Spongin-Like Collagen Scaffolds for Osteoporotic Bone Tissue Engineering

**DOI:** 10.1021/acsomega.5c05580

**Published:** 2025-12-30

**Authors:** Matheus de Almeida Cruz, Karolyne dos Santos Jorge Sousa, Ingrid Regina Avanzi, Amanda Souza, Cintia Cristina Santi Martignago, Mirian Bonifacio, Fernanda Vieira Botelho Delpupo, Mariana Carvalho Simões, Lais Caroline Souza-Silva, Julia Risso Parisi, Livia Assis, Flávia de Oliveira, Marcelo Assis, Renata Neves Granito, E-Liisa Laakso, Ana Cláudia Muniz Rennó

**Affiliations:** † 28105 Federal University of São Paulo (UNIFESP), Department of Biosciences, Santos 11015-020, São Paulo, Brazil; ‡ 199076 Metropolitan University of Santos (UNIMES), Department of Physiotherapy, Santos 11015-020, São Paulo, Brazil; § Brasil University, Post-Graduate Program in Biomedical Engineering, São Paulo 08230-030, São Paulo, Brazil; ∥ 588990 Mater Research Institute − University of Queensland, South Brisbane 4101, Queensland, Australia

## Abstract

Osteoporotic fractures pose a significant clinical challenge due to impaired bone regeneration. In this study, composite scaffolds based on Bioglass 45S5 (BG) and marine-derived spongin-like collagen (SPG) were developed and analyzed. Characterization techniques included scanning electron microscopy (SEM) and Fourier-transform infrared spectroscopy (FTIR) to assess morphology and chemical composition of the components. *In vitro* analyses involved genotoxicity testing by micronucleus assay in CHOK-1 cells and assessment of mineralization potential using Alizarin Red S staining in MC3T3-E1 preosteoblasts. *In vivo* performance was evaluated through implantation in tibial bone defects of ovariectomized rats, followed by histological, histomorphometric, and immunohistochemical analyses at 15- and 30-days postsurgery. The characterization confirmed the successful obtation of the components, with SEM revealing distinct BG particles and fibrillar SPG architecture, and FTIR identifying the presence of key chemical bonds from both components. *In vitro*, BG/SPG scaffolds showed no genotoxic effects and significantly enhanced calcium deposition compared to BG alone, indicating superior mineralization capacity. *In vivo*, the composite scaffolds promoted greater bone regeneration than BG alone, with higher bone volume (BV/TV), increased osteoblast activity (N.Ob/T.Ar and Ob.S/BS), and greater collagen deposition. Immunostaining for RUNX-2 and OPG also confirmed elevated osteogenic activity in the BG/SPG group. These findings demonstrate that BG/SPG scaffolds possess excellent structural integrity, safety, and biological performance, supporting their potential use in bone tissue engineering applications for osteoporotic bone repair.

## Graphical abstract

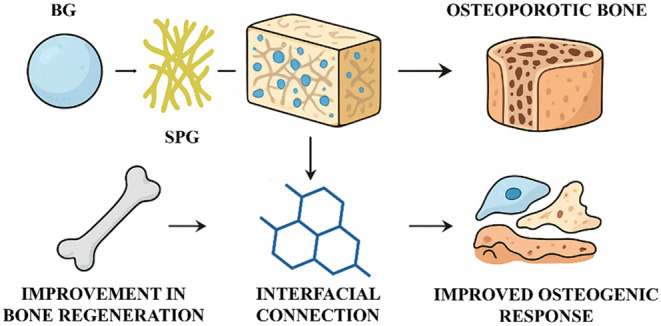

## Introduction

Osteoporosis is a progressive systemic condition marked by a reduction in bone mass and microarchitectural integrity, resulting in increased skeletal fragility and a heightened risk of fractures.^1,2^ These osteoporotic fractures, especially at the hip, vertebrae, and forearm, significantly impact patient independence and impose considerable burdens on healthcare systems worldwide.^3,4^ Beyond their physical consequences, they are often associated with impaired healing, leading to delayed consolidation or nonunion, frequently necessitating surgical intervention.^1^ The development of biomaterials capable of integrating with host bone and stimulating tissue regeneration has thus emerged as a vital strategy to improve clinical outcomes in osteoporotic patients.^5,6^

Among these materials, bioactive glasses (BG), particularly Bioglass 45S5, have long been recognized as gold-standard scaffolds due to their high biocompatibility and capacity to stimulate osteogenesis.^7^ BGs possess a unique surface reactivity, leading to the formation of a hydroxycarbonate apatite (HCA) layer that mimics the mineral component of natural bone and facilitates the recruitment and differentiation of osteoprogenitor cells.^8,9^ Their effectiveness has been demonstrated in both nonload-bearing and load-bearing bone repair strategies.^10,11^ However, their osteoconductive behavior alone may be insufficient in large defects or in compromised metabolic environments such as osteoporosis.^7,12,13^ To overcome these limitations, recent studies have proposed combining BGs with natural biopolymers, especially collagens, to replicate the hierarchical structure of native bone and improve biological functionality.^14–16^

Traditional collagen sources, such as bovine and porcine tissues, raise concerns about zoonotic transmission, immunogenicity, and ethical acceptability.^17,18^ In response, marine-derived collagens have gained traction as sustainable alternatives, offering low immunogenicity, good biocompatibility, and ease of extraction.^19^ In particular, marine sponges (phylum *Porifera*) have emerged as promising candidates due to their unique composition: a mineral phase rich in biosilica (BS) and an organic phase composed of a specialized collagenous protein known as spongin (SPG), which shares structural similarities with type XIII vertebrate collagen.^20,21^ Unlike soluble collagens extracted from fish skin or jellyfish, spongin is an insoluble, highly cross-linked collagen-like protein that forms a stable fibrous network within the sponge skeleton.^22^ Its triple-helical domains are covalently bound to a resilient matrix of halogenated quinone-tanned fibers, which confer remarkable chemical and thermal stability.^23^ This unique structural organization provides superior resistance to enzymatic degradation and supports long-term mechanical integrity, distinguishing spongin from other marine-derived collagens and making its inclusion in composite biomaterials particularly advantageous for bone tissue engineering applications. Recent research by Santos et al. demonstrated that the osteogenic potential of marine sponge-derived scaffolds varies by species, with *Dragmacidon reticulatum* scaffolds promoting significant osteoid tissue formation.^24^ Additional work by Sousa et al. confirmed the biocompatibility and structural adequacy of BS/SPG scaffolds fabricated by 3D printing (BS from *Dragmacidon reticulatum*, and SPG from *Aplysina fulva*), highlighting their potential for bone tissue engineering.^25^

Other investigations have supported the regenerative properties of SPG-based constructs.^26^ For instance, Rennó et al. emphasized the biocompatibility and tissue-regenerative effects of SPG, especially when combined with ceramics.^27^ Sales et al. and Souza et al. demonstrated positive outcomes in skin wound healing when using marine collagen-based dressings, both structurally and at the molecular level, particularly regarding angiogenesis and growth factor expression.^28,29^ Similarly, Cruz et al. showed that SPG combined with BS improved bone formation in an osteoporotic rat model, increasing bone volume fraction and osteogenic marker expression.^30^ These findings collectively underscore the value of SPG as a bioactive, collagenous framework in both dermal and osseous regeneration.

Although the biocompatibility of BG and SPG has been individually confirmed and their combined use has also shown *in vitro* compatibility,^31^ the overall biological performance of the BG/SPG composite remains insufficiently characterized. Notably, the mineralization capacity of BG/SPG scaffolds has not yet been evaluated, despite the critical role of mineral deposition in supporting bone regeneration, particularly in osteoporotic environments characterized by impaired healing.^32^ Equally important, no studies have assessed the genotoxic potential of this composite material. Since long-term safety is essential for any implantable scaffold, evaluating potential DNA damage is fundamental to ensure that these biomaterials do not induce adverse cellular responses.^33^ This gap in literature is especially relevant given the growing demand for multifunctional scaffolds that can simultaneously enhance osteointegration and ensure cytogenetic safety.

Despite this promising body of evidence, the combined use of Bioglass 45S5 and marine sponge-derived spongin in the treatment of osteoporotic bone defects has not yet been thoroughly investigated. We hypothesize that scaffolds composed of BG and SPG will demonstrate superior biological performance compared to BG alone by enhancing bone matrix deposition and accelerating healing in compromised bone. Therefore, this study aimed to characterize the morphology and chemical composition of BG/SPG scaffolds using SEM and FTIR, evaluate their genotoxic profile and mineralization *in vitro*, and assess their biological performance *in vivo* through histological, histomorphometric, and immunohistochemical analyses after implantation into tibial defects in osteoporotic rats at two experimental periods (15 and 30 days). To the best of our knowledge, this is the first study to evaluate Bioglass reinforced with marine-derived spongin-like collagen under osteoporotic conditions, including a comprehensive genotoxicity validation. This highlights the novelty of the work and its translational relevance for bone repair in metabolically compromised environments.

## Materials and Methods

### Bioglass

Amorphous BG, belonging to the system SiO_2_–CaO-Na_2_O–P_2_O_5_,^10^ was produced and provided by Nuclear and Energy Research Institute (particle size: 106–126 μm; IPEN, São Paulo, Brazil).

### Spongin Extraction

SPG was extracted from the marine sponge *Aplysina fulva*. Specimens were collected from two coastal regions in São Sebastião, Brazil: Praia Grande (23°49′23.76″ S, 45°25′01.79″ W) and Araçá Bay (23°81′73.78″ S, 45°40′66.39″ W). This procedure was conducted in accordance with authorization granted by the Brazilian Genetic Heritage Management Council (protocol A92BF77). Upon collection, the samples were rinsed on-site with seawater, stored in seawater-filled containers, and transported to the laboratory. To eliminate cellular debris, the samples were washed three times using Milli-Q water and then stored at −20 °C until processing. The extraction protocol was adapted from the method described by Swatschek et al.^34^ The frozen sponge tissues were cut into smaller fragments and immersed in an extraction buffer composed of 100 mM Tris-HCl (pH 9.5), 10 mM EDTA, 8 M urea, and 100 mM 2-mercaptoethanol. The pH was carefully adjusted to 9 with NaOH, and the mixture was stirred continuously at room temperature for 24 h. Following incubation, the suspension was centrifuged at 5000*g* for 5 min at 2 °C. The pellet was discarded, and the supernatant was retained. To induce precipitation of SPG, the pH was reduced to 4 using acetic acid. The resulting precipitate was washed with Milli-Q water, centrifuged again, and subsequently freeze-dried (lyophilized) for storage and later use.

### Preparation of the Scaffolds

For this study, two types of scaffolds were used: one composed entirely of BG (100% w/w BG), and another consisting of a composite blend of BG and SPG at a ratio of 70% w/w BG and 30% w/w SPG, which closely approximates the natural inorganic-to-organic proportion found in human bone and was previously shown by our group to provide the best balance between mineralization potential and mechanical stability.^16,25^ The materials were loaded into 2 mL plastic syringes along with 0.5 g of sodium phosphate dibasic (Na_2_HPO_4_) powder, which served as the porogenic agent. Subsequently, 1 mL of a 2% Na_2_HPO_4_ aqueous solution was added, and the mixture was homogenized for 20 s using an amalgamator (Silamat, Vivadent, Schaan, Liechtenstein). After mixing, the resulting composite paste was injected into cylindrical molds with dimensions of 0.3 cm in diameter and 0.2 cm in height.

### Scanning Electron Microscopy (SEM)

The samples were fixed onto aluminum stubs using carbon adhesive tape, then coated with a thin layer of gold/palladium using a sputter coater (BAL-TEC MED 020, BAL-TEC, Liechtenstein). Surface morphology was examined using a ZEISS LEO 440 SEM operated at 20 kV with a beam current of 2.82 A.

### Fourier Transform Infrared Spectroscopy (FTIR)

FTIR was used to assess the chemical composition of the materials, employing a PerkinElmer 1700 spectrometer (U.K.). Spectra were acquired in the 400–4000 cm^–1^ range, with a resolution of 2 cm^–1^. Each sample was scanned 100 times, and the resulting spectrum represents the average of those scans.

### pH Evaluation

These analyses were performed in triplicate using BG e BG/SPG (0.1 g/mL). The scaffolds were placed in Falcon tubes with 5 mL of phosphate buffered saline (PBS) and incubated. The electrode of an Orion A211 Star pHmeter (Thermo Scientific, Massachusetts, USA) was then positioned in the Falcon to start the pH measurement from 1 to 14 days.^25^

### Mass Stability

The structures (*n* = 5) were initially weighed to obtain the initial mass and then placed in tubes with 3 mL of PBS and incubated from 1 to 14. The mass loss of the samples was defined as in Equation 1 (eq 1).

1 $$\%\;de\mathrm{grad}ation=\left(W_{f}/W_{i}\right)\times 1000$$

Where *W*_f_ is the weight of the scaffolds after immersion in PBS, and *W*_*i*_ the initial weight of the scaffolds.^35^

### Porosity Evaluation

To determine the porosity, the solvent displacement methodology. Scaffolds (*n* = 8) were initially weighed and then immersed in water for 30 min. To calculate the porosity, the eq 2 was used.

2 $$\mathrm{Porosity}\;\left(\%\right)=\frac{\left(W_{w}-W_{d}\right)\times 100\%}{\left\{\rho \left(\pi R^{2T}\right)\right\}}$$

Where *W*_w_ is the wet weight of the scaffold, *W*_d_ is the dry weight of the scaffold, ρ is the density of the solvent, *R* is the radius of the scaffold, and *T* is the thickness of the scaffold.^25^

### Compression Test

The mechanical properties of the scaffolds were evaluated through uniaxial compression testing using a calibrated Instron universal testing machine equipped with a 50 N load cell. The samples had a cylindrical geometry and were tested in eight independent replicates (*n* = 8). Prior to testing, the dimensions of each specimen were measured using a digital caliper (accuracy ±0.01 mm) to determine the initial cross-sectional area. During the test, each sample was carefully centered between parallel compression plates, and a preload of 0.02 N was applied to ensure proper contact between the surfaces. The displacement was then zeroed, and the test was conducted under displacement control at a crosshead speed of 3 mm/min, corresponding to a strain rate of approximately 0.01 s^–1^ relative to the initial sample height. Compression proceeded until 50% strain or until a maximum load of 45 N was reached.

### Cell Lineages

Rat calvarial preosteoblast cells (MC3T3-E1) and Chinese Hamster Ovary (CHOK-1) cells were used in this study. The selection of cell lines followed the recommendations of ISO 10993-3. MC3T3-E1 cells were cultured in α-MEM, while CHO-K1 cells were maintained in HAM-F12 medium, both supplemented with 10% fetal bovine serum. Cultures were incubated at 37 °C in a humidified atmosphere containing 5% CO_2_. Cells were grown under subconfluent conditions and routinely passaged every 2 to 3 days until they were used in experiments.

### Mineralization Evaluated by Alizarin Red Staining

The analysis of the mineralization potential of the extracts was performed according to Sousa et al.^25^ For this purpose, MC3T3-E1 cells (2 × 10^4^ cells/well) were seeded in a 24-well plate lined with a coverslip. The following experimental groups were established: Mineralization Negative Control (CNM) Cells cultured solely in regular culture medium (α-MEM), mineralization Positive Control (CPM) cells treated with culture medium supplemented with 50 μg/mL of ascorbic acid (AA) and 10 mM β-glycerophosphate (βGP) (α-MEM + AA + βGP), BG Group cells cultured with 100% BG extract (BG + AA + βGP) and BG/SPG Group cells cultured with 100% BG/SPG extract (BG/SPG + AA + βGP). The medium was removed, and the cells were washed with PBS, then fixed in 70% ethanol for 1 h. Subsequently, they were washed with PBS, and the coverslips were stained with Alizarin Red for 15 min. After staining, samples were thoroughly washed with distilled water. Finally, samples were cleared and mounted on glass slides using Permount (Sigma-Aldrich). The presence or absence of mineralization nodules was assessed through qualitative analysis, and photomicrographs were obtained using an Olympus optical microscope (Shinjuku, Tokyo, Japan) at a magnification of 100×, utilizing Olympus software (Shinjuku, Tokyo, Japan).

### Micronucleus Assay

The micronucleus cytome assay was carried out based on the protocol described by Fenech,^36^ with some modifications: use of cytochalasin-B at a concentration of 3 μg/mL, hypotonic solution of 1% sodium citrate at 4 °C, fixation with 25% formaldehyde and staining with rapid panoptic (Newprov, Paraná, Brazil). All experiments were conducted in independent triplicates. In addition to the previously defined groups, a positive control group was included in which the cells were exposed to 40 μM methylmethanesulfonate (MMS) for 4 h. After this period, the cultures were incubated for a further 24 h in the presence of cytochalasin-B, with the aim of blocking cytokinesis and inducing binucleation of the cells. 1. The cells were then treated with trypsin, resuspended in hypotonic solution (1% sodium citrate at 4 °C) for 4 min and centrifuged. The material was then fixed twice with a methanol:acetic acid solution (3:1). After the second fixation, the cells were transferred to previously washed slides and kept in ice-cold distilled water to form the cell film. The samples were stained with rapid panoptic and prepared for microscopic analysis. The evaluation was carried out according to the criteria established by Fenech,^36^ considering the Nuclear Division Index (NDI). For each experiment, 500 cells were analyzed, and only those with intact cytoplasm and containing 1 to 4 nuclei were considered.

### Animals and Experimental Groups

This study was conducted using 60 healthy female Wistar rats, aged 3 months and weighing between 250 and 300 g. This in vivo model was approved by the Ethics Committee on Animal Use CEUA/UNIFESP, registered under number 2629220119. The animals were housed in pairs in standard type 3 cages under controlled environmental conditions and were given a 7-day acclimatization period before any procedure. Osteoporosis was induced through bilateral ovariectomy (OVX), following a well-established experimental protocol.^37^ After a period of 9 weeks to allow for the development of osteoporotic changes, the animals underwent surgery to create a standardized defect in the tibia.

Following the induction of bone defects, the animals were randomly allocated into three groups. The control group consisted of osteoporotic rats that did not receive any scaffold treatment. The second group was treated with scaffolds composed solely of BG, while the third group received composite scaffolds made BG/SPG, respectively. Each group was subdivided according to the time point of evaluation, with euthanasia performed at either 15- or 30-days postsurgery. This resulted in six experimental subgroups, each containing 10 animals, allowing for both early and later-stage analyses of the bone repair process.

### Ovariectomy (OVX)

All animals underwent bilateral ovariectomy (OVX) to induce osteoporosis.^37^ Prior to surgery, general anesthesia was administered via intraperitoneal injection of ketamine (80 mg/kg) and xylazine (8 mg/kg). The abdominal region was shaved and disinfected with iodopovidone. A midline incision was then made using a scalpel blade to access the peritoneal cavity. Both ovaries were located, exteriorized, and surgically removed. Internal closure was performed using absorbable sutures, followed by external suturing with nylon thread. After surgery, the animals were monitored and allowed a 9-week recovery period to ensure the establishment of osteoporotic conditions.

### Experimental Model of Tibia Bone Defect

Nine weeks after the OVX procedure, a standardized bone defect was surgically created in the proximal region of both tibias. Preoperative analgesia was administered 15 min prior to anesthesia induction with an intraperitoneal injection of carprofen (Rimadyl, Pfizer Animal Health, NY, USA) at a dose of 5 mg/kg. Postoperative analgesia was maintained with carprofen every 24 h for a minimum of 2 days. Additionally, buprenorphine (Temgesic, Reckitt Benckiser, U.K.) was administered every 12 h at a dose of 0.01 mg/kg. Anesthesia was induced and maintained using isoflurane delivered by mask in combination with oxygen. The surgical site, on the medial aspect of the right tibia, was shaved and disinfected with povidone-iodine. Animals were placed in the supine position, and a 10 mm longitudinal incision was made through the skin and underlying muscle. A circular bone defect (3 mm in diameter) was drilled into the proximal third of the tibia, approximately 10 mm distal to the knee joint, using a motorized drill (Beltec, Araraquara, SP, Brazil) under constant irrigation with sterile saline to minimize thermal damage. The scaffold material was inserted into the defect, and the wound was closed with layered sutures. Before completing the closure, local anesthesia was applied directly to the site using a mixture of lidocaine (10 mg/mL) and bupivacaine (5 mg/mL), diluted in saline, with approximately 1 mL administered per animal (equivalent to 1 mg lidocaine and 0.25 mg bupivacaine). Animals were euthanized at the designated time points (15- and 30-days postsurgery) using an overdose of ketamine (240 mg/kg) and xylazine (24 mg/kg).

### Histopathological Analysis

Following euthanasia, the tibias were carefully dissected and immediately fixed in 10% buffered formalin (Merck, Darmstadt, Germany) for 48 h. Samples were then decalcified in 4% ethylenediaminetetraacetic acid (EDTA) solution (Merck, Darmstadt, Germany) and subsequently dehydrated through a graded ethanol series. After dehydration, specimens were embedded in paraffin blocks for histological processing. Serial sections of 5 μm thickness were obtained using a rotary microtome equipped with a disposable blade (Leica SP1600, Leica Microsystems, Nussloch, Germany). The sections were mounted on glass slides, stained with hematoxylin and eosin (H&E, Merck, Darmstadt, Germany), and examined under a light microscope (Leica Microsystems AG, Wetzlar, Germany). For each sample, the presence of granulation tissue, areas of newly formed bone, residual biomaterial, and the condition of the bone defect margins were assessed. Histological evaluation was performed independently by two blinded observers to ensure unbiased interpretation of the findings.

### Histomorphometric Analysis

Quantitative histomorphometric analysis was carried out using the semiautomated OsteoMeasure System (Osteometrics, Atlanta, GA, USA). This system allowed for the assessment of structural parameters related to bone formation and residual biomaterial within the defect area. Each specimen was analyzed individually to enable comparison across experimental groups. The following parameters were evaluated: bone volume fraction (BV/TV), number of osteoblasts per tissue area (N.Ob/T.Ar), and the percentage of bone surface covered by osteoblasts (Ob.S/BS). All measurements were performed in a blinded manner by two independent, experienced observers to ensure consistency and reduce bias in the data analysis.

### Picrosirius Staining

Histological sections of the tibia were stained with Sirius Red and examined under polarized light to distinguish between collagen type I fibers (appearing yellow to red) and type III fibers (appearing green). The volume fraction of each collagen type (Vv) and the total collagen volume fraction (Vv_Total_) were calculated. For this analysis, ten photomicrographs per animal were captured from the region of newly formed bone using a digital imaging system (AxioVision 4.5, Zeiss) connected to a microscope (Axio Observer D1, Zeiss) equipped with a 40× objective. Each image was analyzed using a test system containing 36 randomly positioned crosses, and the assessment was performed in a blinded manner. Collagen fibers that intersected the upper quadrant of each cross were counted. For each photomicrograph, the total number of intersection points over the area of interest (*P*_t_) and the number of points intersecting collagen type I, type III, or both (*P*_collagen_) were determined.

The volume fraction of each collagen type was calculated using the following eq 3

3 $$V\_V_{\mathrm{collagen}}=\left(\left(\sum P\_\left(\mathrm{collagen}\;\mathrm{type}\;I,\mathrm{II}\;\mathrm{or}\;\mathrm{total}\right)\right)/\left(\sum P_{t}\right)\right)\times 100$$

This method allowed for a quantitative estimation of the proportion of each collagen fiber type within the newly formed bone tissue.^38^

### Immunohistochemistry Analysis

The streptavidin–biotin–peroxidase technique, as previously reported by Magri et al.^39^ was used to perform immunohistochemical analysis. The tissue slices were first deparaffinized with xylene and then rehydrated with a series of graded ethanol. Sections were heated in 0.01 M citric acid buffer (pH 6.0) with a steamer for 5 min to perform antigen retrieval. The slides were incubated in a hydrogen peroxide solution in phosphate-buffered saline (PBS) for 5 min to inhibit endogenous peroxidase activity. Endogenous peroxidase activity was blocked by incubating the slides in a solution of hydrogen peroxide in phosphate-buffered saline (PBS) for 5 min. After that, nonspecific binding sites were blocked for 10 min using 5% normal goat serum in PBS. The primary antibody was incubated at 4 °C for the whole night. The antibodies used were a polyclonal anti-RUNX-2 (code: sc-8566, Santa Cruz Biotechnology, USA) at a dilution of 1:100, and a monoclonal antiosteoprotegerin (OPG) (code: sc-7269, Santa Cruz Biotechnology, USA). After rinsing, the sections were incubated with a biotinylated secondary antirabbit IgG antibody (Vector Laboratories, Burlingame, CA, USA) at a dilution of 1:200 in PBS for 1 h. This was followed by incubation with an avidin–biotin–peroxidase complex for 45 min. A 0.05% solution of 3,3′-diaminobenzidine (DAB) was used to observe immunostaining, and Harris hematoxylin (Merck) was used for 10 s of counterstaining. Lastly, qualitative analyses were conducted to assess the presence and distribution of the immunomarkers using light microscopy (Leica Microsystems AG, Wetzlar, Germany). In addition, semiquantitative scoring was applied as follows: 1 = absent (0% immunostaining), 2 = weak (1–25%), 3 = moderate (36–67%), and 4 = intense (68–100%) immunostaining. All evaluations were performed in a blind manner by two independent observers (M.A.C. and A.S.)

### Statistical Analysis

Quantitative data were presented as mean ± standard deviation. Statistical analyses were conducted using GraphPad Prism version 9 (GraphPad Software, San Diego, CA, USA). The Shapiro–Wilk test was applied to assess the normality of data distribution. For data sets not following a normal distribution, nonparametric tests were used, including the Mann–Whitney test for two-group comparisons and the Kruskal–Wallis test followed by Dunn’s post hoc test for multiple comparisons. For normally distributed data, comparisons were performed using the unpaired Student’s *t* test or two-way analysis of variance (ANOVA) followed by Tukey’s multiple comparisons test. A significance level of *p* ≤ 0.05 was adopted for all analyses.

## Results

### Scanning Electron Microscopy (SEM)

Figure 1 shows the scanning electron microscopy (SEM) images of BG and spongin-like collagen (SPG), analyzed individually. In Figure 1A,B, BG particles appear dense, with irregular and angular shapes, and a smooth surface. In contrast, Figure 1C,D display the morphology of SPG, which presents a fibrillar microstructure with clustered fibers and a porous, uneven arrangement. These images clearly illustrate the distinct structural features of each material, which are relevant to their function when combined into composite scaffolds.

**Figure 1:**
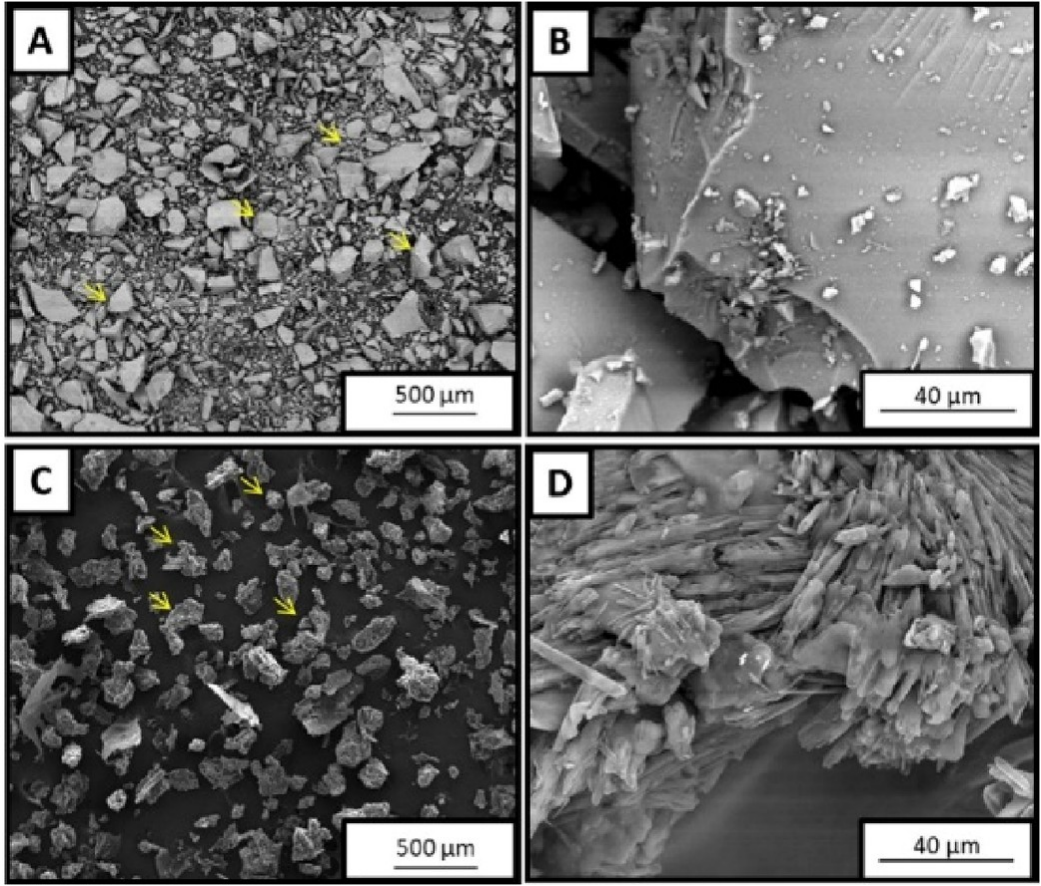
SEM photomicrographs of BG smooth surface irregular particles (indicated by arrows) of BG in 100× (A) and 2000× (B); fibrillar granules of SPG (indicated by arrows) in 100× (C) and 2000× (D).

### Fourier Transform Infrared Spectroscopy (FTIR)

Figure 2 presents the FTIR spectra obtained for the individual components used in scaffold fabrication: BG and SPG. In the spectrum of BG (Figure 2A), characteristic bands were identified at 912 cm^–1^, corresponding to Si–O stretching vibrations, and at 788 cm^–1^, attributed to P–O bending vibrations, both of which are typical of silicon oxide and phosphate groups present in BG.^40^ These peaks confirm the structural integrity and chemical composition of the BG used in this study. The spectrum of SPG (Figure 2B) revealed distinct absorption bands associated with functional groups commonly found in collagen-based materials.^41^ Notably, a broad band at 3362 cm^–1^ is attributed to N–H stretching vibrations, while the peak at 1594 cm^–1^ corresponds to N–H bending, both confirming the presence of amine groups.^30^ In addition, multiple peaks were observed in the 1000–1500 cm^–1^ region, which are related to C–O stretching vibrations, further supporting the collagenous nature of the organic matrix.^26^ These spectral features validate the chemical identity of SPG as a proteinaceous material and support its role as a bioactive and biocompatible component in the composite scaffolds.

**Figure 2:**
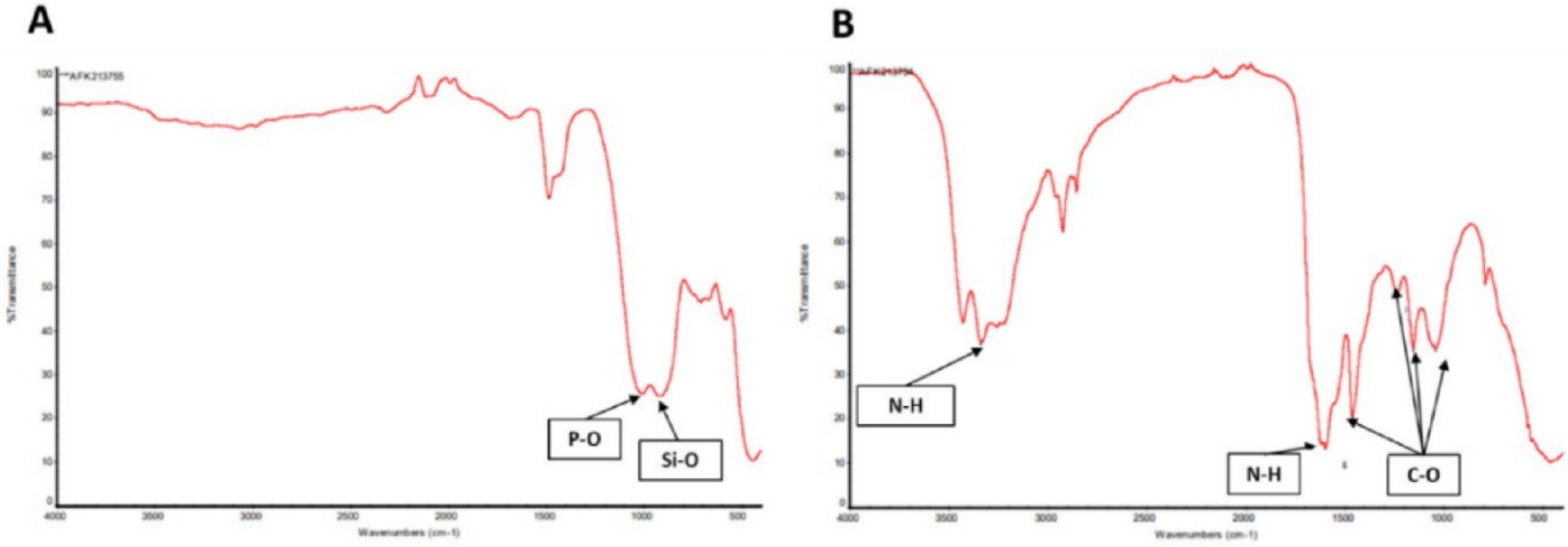
FTIR spectrum of BG (A) and SPG (B).

### pH Evaluation

The pH evaluation of the BG and BG/SPG samples during 14 days of immersion in an aqueous medium revealed progressive alkaline behavior in both groups, with a marked increase already in the first 24 h. Initially, both materials presented values close to 6.77, indicating a slightly acidic neutral medium. After 24 h, a significant increase in pH to values around 12.9 was observed in both groups, characterizing the rapid release of alkaline ions resulting from ion exchange between the surface of the bioactive glass and the medium. In the following days, pH values remained high, stabilizing between 13.3 and 14.0, with small variations until the 14th day. The BG group tended to reach values slightly higher than those of the BG/SPG group, reaching a plateau of approximately 14.0 from the eighth day onward, while the BG/SPG group maintained an average pH close to 13.6 in the same period (Figure 3).

**Figure 3:**
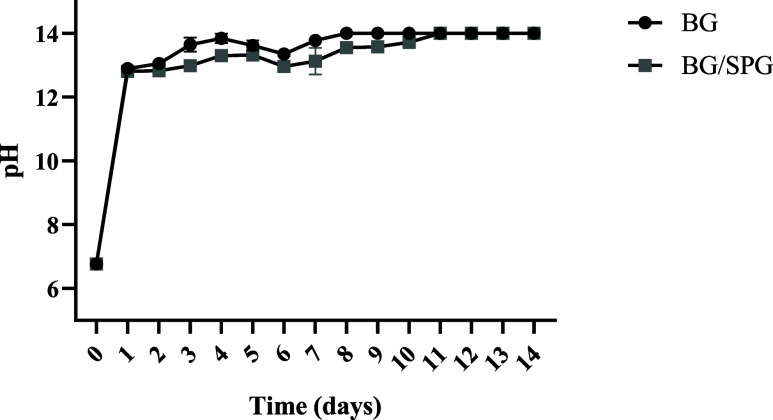
pH variation of BG and BG/SPG samples during 14 days of immersion.

### Mass Stability

The analysis of mass stability revealed distinct behaviors between the BG and BG/SPG groups over the 14 days of immersion. Both groups showed an initial increase in mass in the first 48 h, indicating fluid absorption and possible ion deposition on the surface of the materials. However, this increase was more pronounced in the BG/SPG group, which reached average values greater than 150% of the initial mass, while the BG group showed a moderate increase, reaching about 120% in the same period. After this initial peak, a gradual reduction in mass was observed in both groups, associated with the onset of dissolution and surface restructuring of the materials. The BG group showed a continuous loss over time, stabilizing at around 90% of the initial mass on the 14th day. The BG/SPG group, on the other hand, maintained higher and more stable values, with approximately 120% of the initial mass at the end of the experimental period (Figure 4).

**Figure 4:**
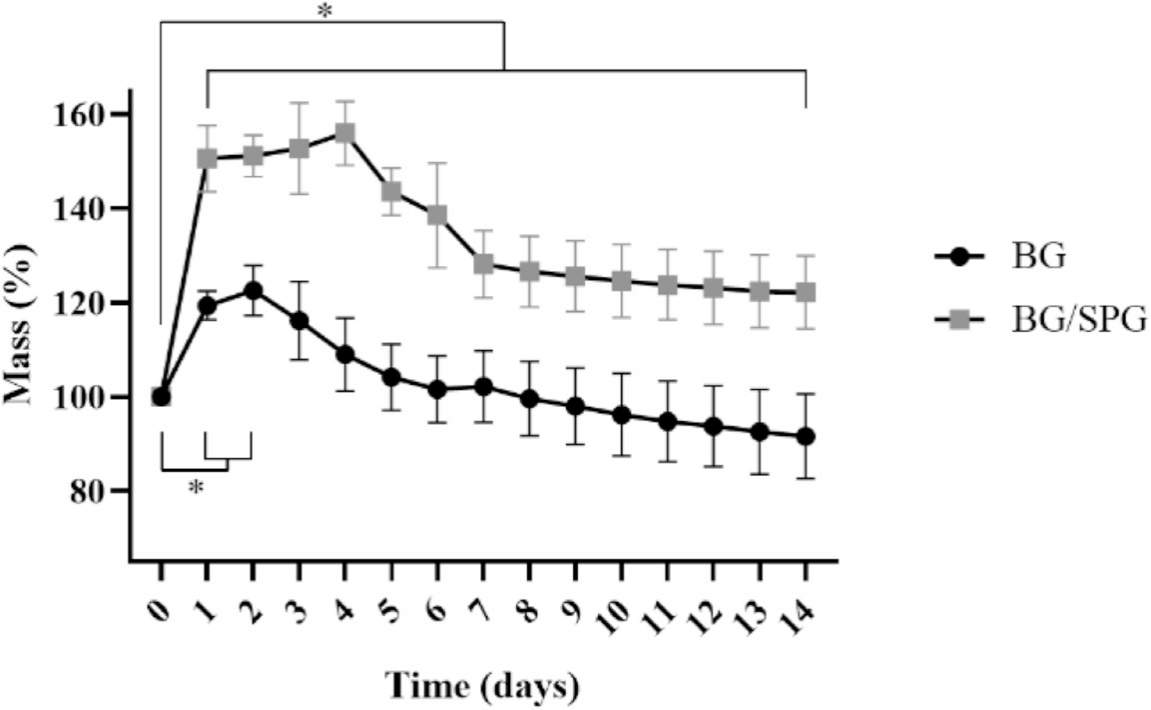
Mass stability of BG and BG/SPG samples over 14 days of immersion.

### Porosity Evaluation

The porosity of the scaffolds is presented in Table 1, showing that the BS sample exhibited an average porosity of 59.8 ± 13.2%, while the BS/SPG sample reached 72.5 ± 4.5%. This result indicates that the incorporation of spongin led to a significant increase in porosity, promoting a more open and interconnected network that may enhance nutrient diffusion and cell migration within the scaffold.

**Table 1:** Porosity, Compressive Strength (σ), and Elastic Modulus (*E*) of the Samples^a^

Sample	Porosity (%)	σ (Pa)	*E* (MPa)
BS	59.82 ± 13.2	128.93 ± 38	4.69 ± 1.21
BS/SPG	72.51 ± 4.5*	758.60 ± 59*	9.32 ± 1.83*

a Values are expressed as mean ± SD (*n* = 8). *p < 0.05.

### Compression Test

The mechanical properties of the scaffolds are presented in Table 1, showing that the BS sample exhibited a compressive strength (σ) of 128.9 ± 38 Pa and an elastic modulus (*E*) of 4.69 ± 1.21 MPa, whereas the BS/SPG sample reached 758.6 ± 59 Pa and 9.32 ± 1.83 MPa, respectively. These results demonstrate that the addition of spongin led to a remarkable increase in both strength and stiffness, indicating enhanced integration between the organic and inorganic phases and a more efficient stress distribution within the scaffold structure.

### Mineralization Evaluated by Alizarin Red Staining

Alizarin Red S staining is a common method used to identify calcium-rich deposits as an indicator of extracellular matrix mineralization. Figure 5 presents the *in vitro* results of Alizarin Red S staining performed on MC3T3-E1 preosteoblastic cells after 7 and 14 days of treatment. This assay was used to assess the mineralization potential of the different scaffolds. In the negative control group (CNM), no calcium deposits were detected at either time point, as evidenced by the absence of staining (Figure 5A,B). In the positive control group (CPM), slightly and localized mineral deposition was observed after 7 days (Figure 4C), which progressed to more pronounced and scattered staining by day 14 (Figure 5D). In the BG group, intense staining was already evident on day 7 (Figure 5E), indicating early mineralization activity, which appeared comparable to the CPM group by day 14 (Figure 5F). The composite group containing BG/SPG exhibited markedly stronger Alizarin Red staining than the CPM group at both 7 and 14 days (Figure 5G,H), suggesting a greater capacity to promote extracellular matrix mineralization under osteogenic conditions.

**Figure 5:**
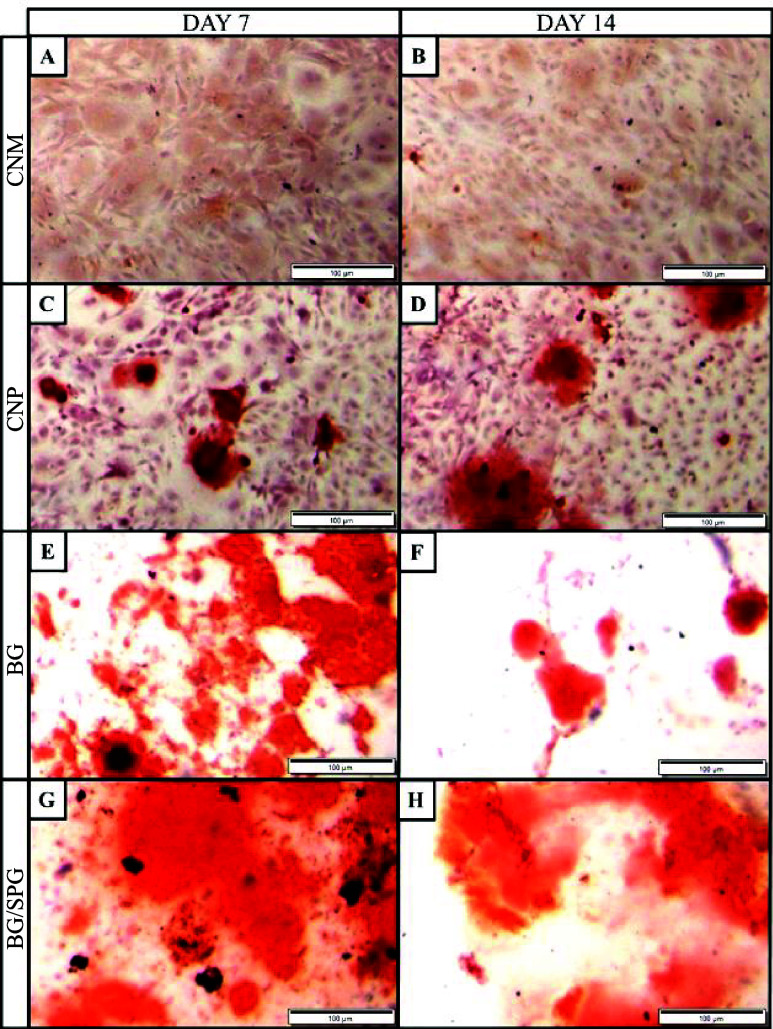
Alizarin Red staining at 100× magnification. CNM 7 days (A) and 14 days (B). CPN 7 days (C) and 14 days (D). BG 7 days (E) and 14 days (F). BG/SPG 7 days (G) and 14 days (H).

### Micronucleus

Figure 6 shows the results of the micronucleus assay conducted on CHO-K1 cells after 4 h of exposure. As expected, all concentrations of MMS, used as a positive control, resulted in a significantly higher frequency of micronuclei when compared to the negative control group (GC) and the experimental groups treated with BG and BG/SPG scaffolds. No statistically significant differences were observed between GC, BG, and BG/SPG groups, indicating that exposure to the tested scaffold did not induce genotoxic effects under the experimental conditions.

**Figure 6:**
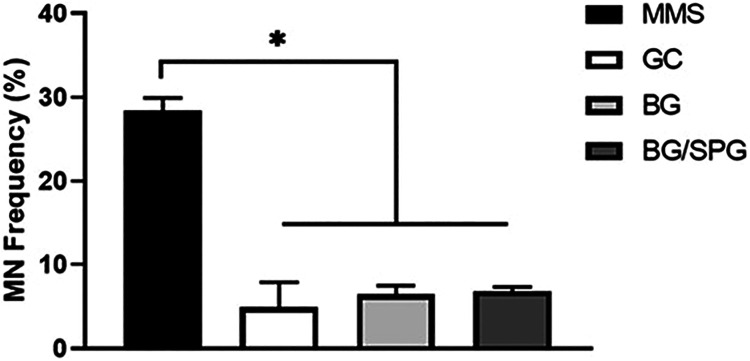
Micronucleus results for CHOK-1. * *p* < 0.0001. ANOVA test followed by post hoc by Tukey.

### Histopathological Analysis

Figure 7 presents the histological findings of the experimental periods at 15- and 30-days postsurgery.

**Figure 7:**
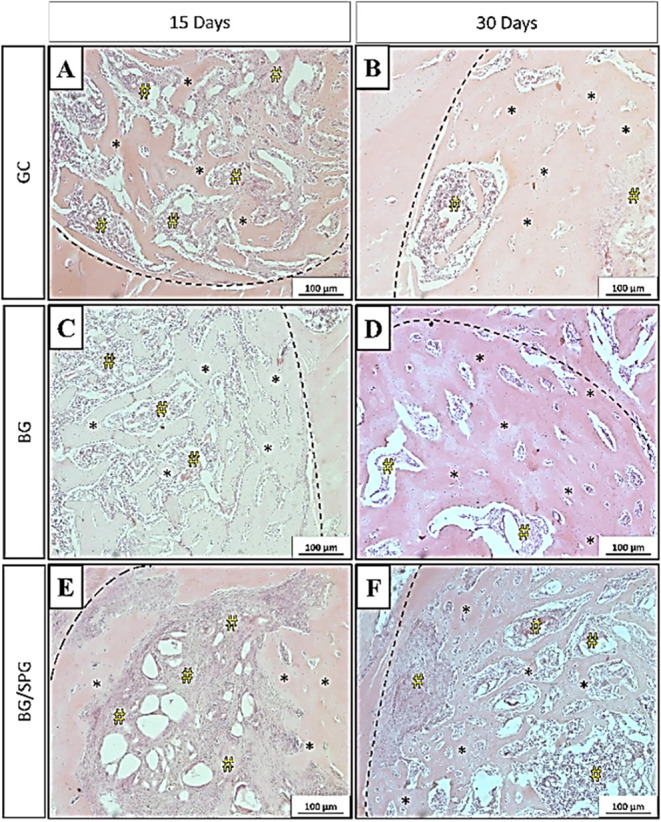
H&E-stained histological results at 10× magnification. GC 15-days postsurgery (A) 30-days postsurgery (B); BG 15-days postsurgery (C) 30-days postsurgery (D); BG/SPG 15-days postsurgery (E) 30-days postsurgery (F). Bone tissue (*), granulation tissue (#) and defect border (dashed line).

Fifteen days after surgery, histological evaluation at 20× magnification revealed that the bone defect borders in the control group (CG) were still clearly defined. Granulation tissue occupied the central region of the defect, and newly formed bone was observed at the periphery (Figure 7A). In the group treated with BG, the defect margins remained visible, and the central area was also predominantly filled with granulation tissue. Newly formed bone tissue and residual biomaterial particles were present at the outer edges of the defect (Figure 7C). In animals treated with the BG/SPG composite, the borders of the defect were still distinguishable. The central area contained both granulation tissue and remaining scaffold material, while bone formation was evident along the periphery (Figure 7E).

At 30 days postsurgery, the control group continued to show identifiable defect borders, though they were less distinct compared to the earlier point. Granulation tissue was still present in parts of the defect, and bone tissue had begun to grow from the periphery toward the center (Figure 7B). In the BG group, the defect was largely filled with newly formed bone, with some biomaterial particles still visible, along with signs of degradation and residual granulation tissue in the central region. The amount of bone formation had increased considerably compared to day 15 (Figure 7D). The BG/SPG group showed a similar pattern, with less defined defect edges and visible remnants of the biomaterial. Bone regeneration appeared more advanced, with granulation tissue and new bone occupying the central portion of the defect and formation progressing from the edges inward (Figure 7F).

### Histomorphometry

Figure 8 presents the results of the histomorphometric analysis. At 15 days postsurgery, no statistically significant differences in bone volume fraction (BV/TV, %) were observed between the groups. However, after 30 days, the BG/SPG group showed significantly higher BV/TV values compared to both BG and control groups (*p* < 0.0001) (Figure 8A). Regarding the percentage of bone surface covered by osteoblasts (Ob.S/BS, %), BG/SPG also demonstrated a significantly greater value than BG and control at 15 days (*p* < 0.0001), and this difference remained significant at 30 days, with BG/SPG showing higher values compared to BG (*p* < 0.001) and control (*p* < 0.0001) (Figure 8B). As shown in Figure 8C, the number of osteoblasts per tissue area (N.Ob/T.Ar, mm^2^) was significantly higher in the BG/SPG group compared to BG (*p* < 0.0001) and control (*p* < 0.001) at the 15-day time point. Additionally, in the intragroup comparison, BG/SPG showed increased values at 30 days compared to the control group (*p* < 0.01). No other significant differences were found among the groups for the evaluated parameters.

**Figure 8:**
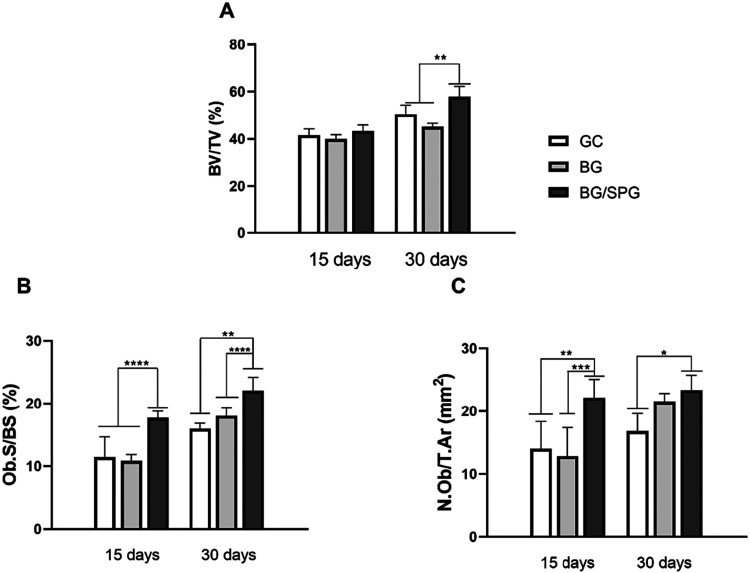
Means and SD of the histomorphometry (% BV/TV (A), % OB.S/BS (B), and N.Ob/T.Ar (C)). Dunn test (*p < 0.0001, **p < 0.01, ***p < 0.001 e ****p < 0.0001).

### Picrosirius Staining

Figure 9 shows the distribution of collagen fibers in the different experimental groups. At 15 days postsurgery, the control group (GC) exhibited collagen fiber networks in the newly formed bone areas, particularly surrounding the trabecular structures. In the BG and BG/SPG groups, the presence of collagen fibers was also evident within the defect area, indicating active extracellular matrix remodeling (Figure 9A,C,E). By day 30, all groups displayed more mature and organized collagen structures. Collagen fibers appeared denser and arranged in parallel bundles, especially in the GC group, reflecting progressive matrix stabilization and tissue maturation (Figure 9B,D,F).

**Figure 9:**
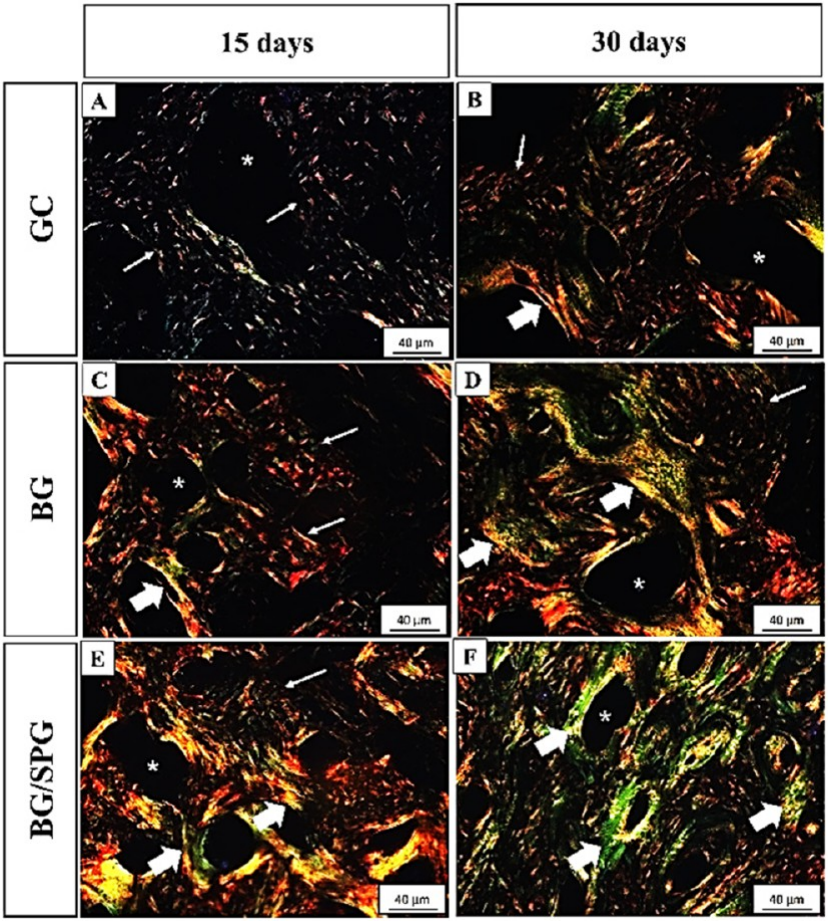
Tibial bone photomicrographs of newly formed bone: GC 15-days postsurgery (A) 30-days postsurgery (B); BG 15-days postsurgery (C) 30-days postsurgery (D); BG/SPG 15-days postsurgery (E) 30-days postsurgery (F). Stained with Sirius-Red and analyzed under polarized light, the images show the differentiation between collagen fibers type I (yellow and red) and collagen fibers type III (green) in trabecular bone regions. Note the networked collagen arrangement (thin arrows) predominant in the first 15-day period compared with denser arrangement (thick arrows) after 30 days. Asterisk = bone marrow. Scale Bar = 40 μm.

Figure 10 presents the quantitative analysis of collagen fiber content across groups and time points. At 15 days postsurgery, both BG and BG/SPG scaffolds exhibited a significantly higher volume fraction (Vv) of type I collagen fibers when compared to the control group (GC) (*p* < 0.0001), as shown in Figure 10A. However, by day 30, no statistical differences were observed among the groups for this parameter. Regarding type III collagen (Figure 10B), the BG/SPG group showed the highest Vv values at 15 days, with statistically significant differences compared to both BG and GC (*p* < 0.0001). Additionally, GC displayed greater type III collagen content than BG alone (*p* < 0.0001). At the 30-day time point, no differences were noted among the experimental groups. For the total collagen volume fraction (Vv_Total_), BG/SPG demonstrated significantly higher values than GC at 15 days (*p* < 0.001), while no significant difference was detected between BG and BG/SPG. Again, no differences were found at 30 days postsurgery (Figure 10C).

**Figure 10:**
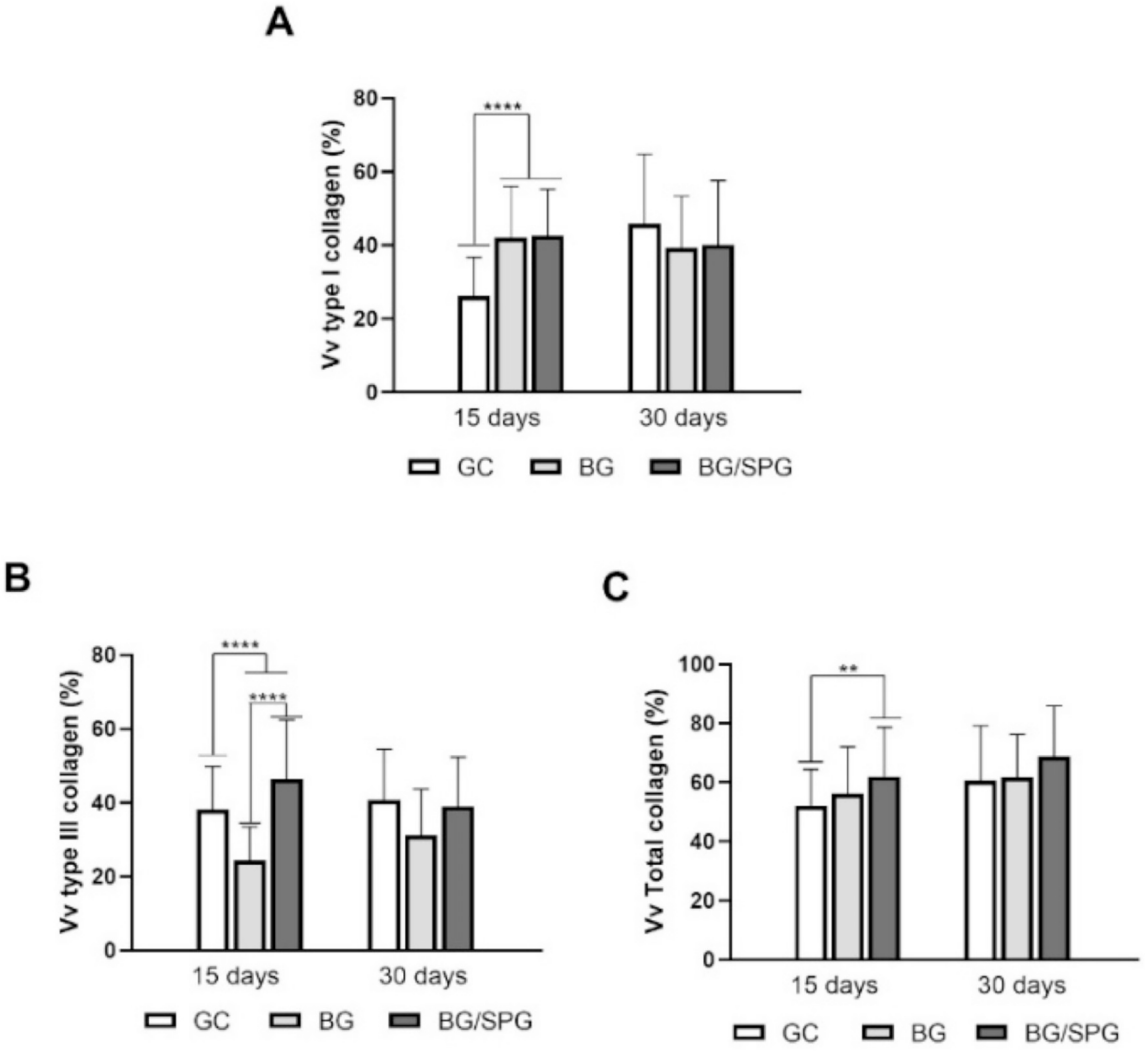
Mean and SD of the Vv % Collagen type I (A), % of type III (B) and % Vv of total Collagen (C). Kruskal–Wallis for intergroups evaluation, followed by post hoc (Parwaise) and Bonferroni Mann–Whitney for intragroup evaluation. (****p < 0.001 and **p < 0.001).

### Immunohistochemistry

Figure 11 illustrates the qualitative distribution of RUNX-2 immunostaining among the experimental groups. In the control group (GC), immunoreactivity for RUNX-2 was primarily localized within granulation tissue at 15 days postsurgery (Figure 11A), whereas at 30 days, staining became more prominent in areas of newly formed bone (Figure 11B). In the BG group, RUNX-2 expression was noted around biomaterial particles and within granulation tissue at both time points. By 30 days, positive staining also extended into the newly formed bone matrix (Figure 11C,D). In the BG/SPG group, RUNX-2 was consistently detected across both granulation tissue and regions of new bone formation at 15 and 30 days (Figure 11E,F), indicating a broader activation of osteogenic pathways in response to the composite scaffold. Semiquantitative analysis of RUNX-2 immunostaining after 15- and 45-days postsurgery was shown in Figure 12. No difference among the experimental groups was observed in the first (*p* = 0.7951) and second (*p* = 0.6847).

**Figure 11:**
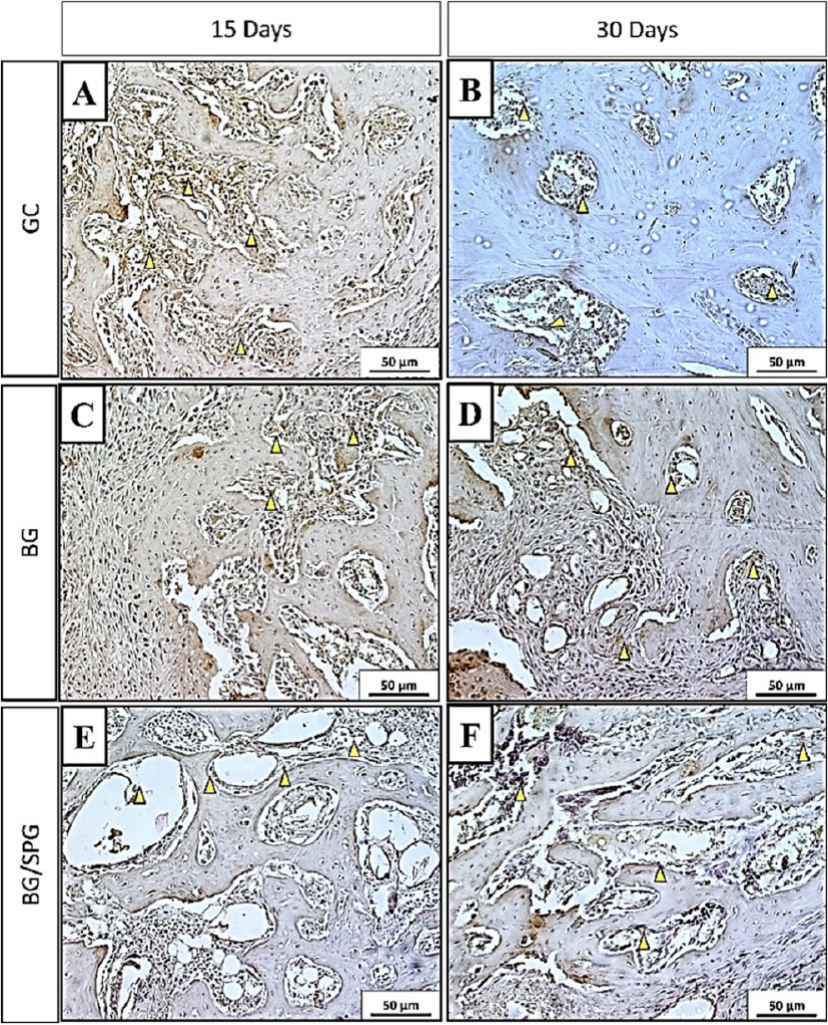
RUNX 2 immunohistochemical analysis. CG 15-days postsurgery (A); 30-days postsurgery (B); BG 15-days postsurgery (C) 30-days postsurgery (D); BG/SPG 15-days postsurgery (E) 30-days postsurgery (F). Magnitude 20×.

**Figure 12:**
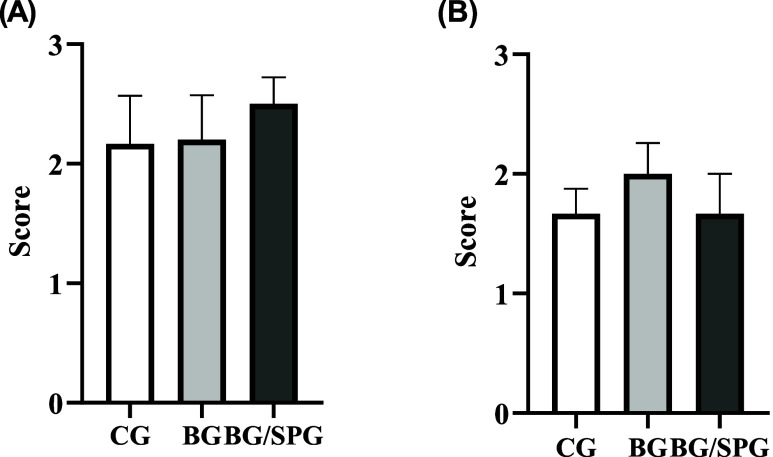
Representative results of the semiquantitative immunohistochemistry score evaluation for RUNX-2 at 15 (A) and 45 (B) days postinjury. (*p* > 0.05; Kruskal–Wallis test; media ± SEM; *n* = 6).

Figure 13 presents the qualitative assessment of OPG immunostaining across the different groups. In the control group (GC), OPG expression was primarily detected in granulation tissue at 15 days postsurgery and later localized within areas of newly formed bone at 30 days (Figure 13A,B). In the BG group, immunostaining was evident within granulation tissue and early bone matrix at 15 days (Figure 13C), while by 30 days, OPG expression became more prominent in the newly formed bone and in the vicinity of residual BG particles (Figure 13D). In animals treated with BG/SPG scaffolds, OPG staining at 15 days was observed predominantly around BG fragments and in granulation tissue regions. After 30 days, the immunostaining pattern extended throughout the bone trabeculae and remaining granulation tissue (Figure 13E,F), suggesting progressive involvement in bone remodeling and maturation. Semiquantitative analysis of OPG immunostaining after 15- and 45-days postsurgery was shown in Figure 14. No difference among the experimental groups was observed in the first (*p* = >0.9999) and second (*p* = 0.4422).

**Figure 13:**
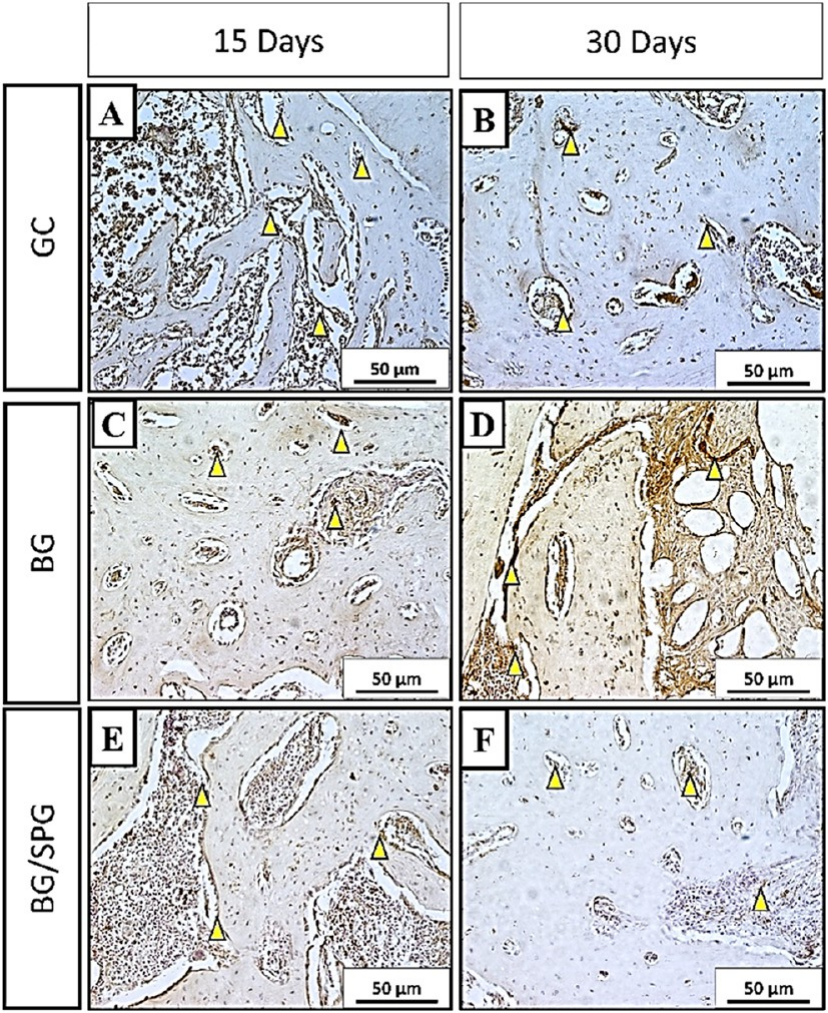
Immunohistochemical analysis of OPG. GC 15-days postsurgery (A); 30-days postsurgery (B); BG 15-days postsurgery (C) 30-days postsurgery (D); BG/SPG 15-days postsurgery (E) 30-days postsurgery (F). Magnitude 20×.

**Figure 14:**
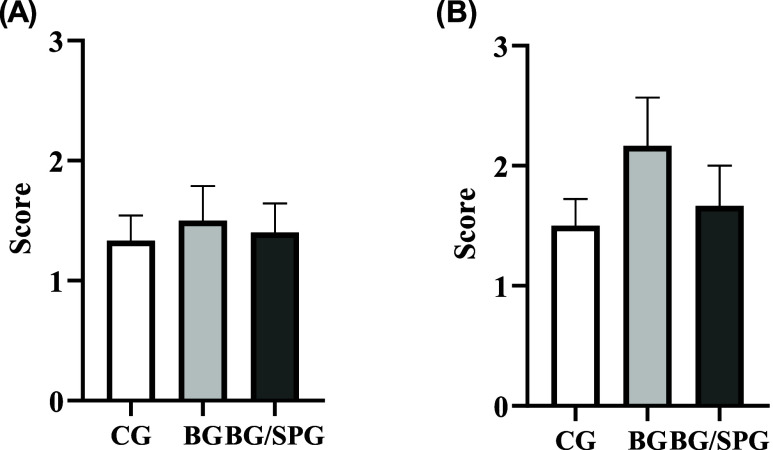
Representative results of the semiquantitative immunohistochemistry score evaluation for OPG at 15 (A) and 45 (B) days postinjury. (*p* > 0.05; Kruskal–Wallis test; media ± SEM; *n* = 6).

## Discussion

The aim of the present study was to characterize the physicochemical properties of BG and SPG, assess their mineralization and genotoxicity potential *in vitro*, and evaluate the *in vivo* biological effects of BG and BG/SPG scaffolds in a tibial defect model using osteoporotic rats. SEM morphological analysis confirmed the successful synthesis of both BG and SPG. BG particles exhibited varied sizes with irregular and angular shapes, along with a smooth surface, while SPG displayed a granular appearance characterized by a fibrillar and irregular microstructure. Irregular granules of BG were also shown by Gabbai-Armelin et al.^42^ and fibrillar aspect of SPG was also observed by Parisi et al.^43^ Regarding chemical composition, the FTIR results supported the successful identification of both materials. For BG, characteristic peaks of silicon oxide and phosphorus oxide were observed, in agreement with the findings reported by Gabbai-Armelin et al.^44^ In the case of SPG, the presence of −NH_3_ and −CO groups corroborated the data previously described by Santana et al.,^45^ confirming its proteinaceous and collagen-like nature. The proposed volume to prepare BG/SPG composite (70:30 w/w) used in the present study was inspired to mimic the natural bone composition.^44^

The stability and mechanical results demonstrate that the addition of spongin produced notable effects on the physical, chemical, and mechanical behavior of the scaffolds. Both samples showed a rapid increase in pH during the first 24 h, typical of ion exchange from bioactive glass,^46^ but the BG/SPG group maintained slightly lower values, suggesting a more controlled release of alkaline species. Mass variation followed a similar trend: while both groups initially absorbed fluid, the BG/SPG samples exhibited greater stability, retaining a higher percentage of their initial mass after 14 days. The higher porosity observed for the BG/SPG group (72.5 ± 4.5%) was accompanied by a substantial improvement in compressive strength and stiffness, indicating that spongin contributed to a more homogeneous structure capable of supporting higher mechanical loads. A similar increase in porosity associated with spongin incorporation was also reported by Sousa et al.^25^ in a previous study, reinforcing its role in promoting a more open and interconnected architecture within collagen-based composites. Overall, the incorporation of spongin enhanced the balance between porosity, stability, and mechanical performance, which is desirable for scaffolds intended for bone regeneration.

The Alizarin Red S assay revealed significantly enhanced mineralization potential in the BG/SPG group compared to BG alone and the positive control, suggesting a synergistic effect between BG and SPG. While previous studies such as Sousa et al. attributed mineralization primarily to the BS component in BS/SPG scaffolds,^25^ and Fernandes et al. reported no increase in early osteoblast differentiation with SPG addition based on ALP activity,^31^ the present findings diverge by demonstrating that SPG, when combined with BG, may act as a favorable organic matrix that enhances nucleation and deposition of calcium-rich nodules. This synergy is likely due to the complementary roles of BG in ion release and SPG in providing a collagenous scaffold, resulting in more pronounced and widespread mineral deposition under osteogenic conditions.

No genotoxic effects were detected for either BG or BG/SPG scaffolds, as demonstrated by the micronucleus assay performed on CHO-K1 cells. The lack of increased micronuclei formation across all experimental groups reinforces the biosafety of these materials for biomedical applications, particularly in bone regeneration. Similar results were reported by Santana et al., who evaluated spongin-rich scaffolds derived from *Aplysina fulva* in combination with bovine collagen and found no genotoxicity.^45^ Likewise, Sousa et al. demonstrated that BS/SPG composites did not raise micronucleus frequency, supporting the genetic safety of SPG-based biomaterials.^25^ Regarding BG, Kido et al. used the comet assay and found no evidence of DNA damage in BG-containing scaffolds,^47^ consistent with the findings of Souza et al., who also reported no genotoxic effects using the micronucleus test.^48^ Together, these results strengthen the evidence that BG/SPG composites are not only bioactive and regenerative but also genetically safe, highlighting their potential for clinical translation.

BG is a bioactive glass material widely used in biomedical research, and it has shown an important role in the enhancement of new bone formation due to its bioactivity and osteoconduction.^49^ Some authors have demonstrated that SPG is able to promote bone cell proliferation and bone healing in healthy animals.^45,50^ To progress the investigation of the effects of BG and BG/SPG scaffolds in the process of bone healing, in the present study, a model of tibial bone defect in osteoporotic rats was used. Histological analysis demonstrated that BG and BG/SPG treated animals showed no adverse reaction, with an absence of material rejection and lack of inflammatory exacerbation, which indicates that the composites were biocompatible,^45,51^ also it is observed that SPG scaffolds evoke no inflammatory response in an animal model of tibial bone defects, confirming evidence of biocompatibility.

Biomaterial degradation was observed through experimental periods, which is an important process that promotes BG ion release,^52,53^ which may have contributed to the osteogenic and osteoconductive response observed in the BG/SPG group, suggesting an indirect role of ion release in modulating the local environment conducive to bone formation.^54^ The histomorphometry showed higher values for BG/SPG compared to BG-treated animals, demonstrating that combining materials into composites promoted the increase in bone volume and in the number of osteoblasts at the site of the defect.^55^ These results are consistent with the findings from the Alizarin Red assay performed *in vitro* using MC3T3-E1 cells, where the BG/SPG scaffolds significantly enhanced mineralization compared to both the control group and the scaffolds composed solely of BG. Many authors have demonstrated the addition of an organic part such as SPG into inorganic materials constitutes a biomimetic composite, similar to bone tissue, becoming a biomaterial with enhanced biological performance.^25,56,57^ The release of ions (including Ca and Si) from BG samples, forms a calcium phosphate layer, consequently attracting osteoprogenitor cells and increasing the rate of bone formation into BG-based granular material.^58^ The improved osteogenic and mechanical performance observed for BG/SPG scaffolds may also be related to interfacial bonding between the two phases. The amino and carboxyl groups in spongin can chelate Ca^2+^ ions released from Bioglass, facilitating the nucleation of hydroxycarbonate apatite and creating a chemically integrated interface.^59^ This interaction not only enhances mechanical cohesion within the scaffold but also promotes sustained ion exchange and biological signaling, providing a favorable microenvironment for osteoblast differentiation and matrix mineralization.^60^

Some authors have demonstrated the positive influence of BG and collagen on the process of bone healing.^61^ Nijsure et al. demonstrated that BG/Collagen based scaffolds enriched with copper produced osteoblast growth and attachment, being a promising alternative for bone tissue engineering purposes.^62^ Collagen type I is responsible for mechanical and structural properties, acting like a natural structure for extracellular matrix proteins synthesis which contributes for bone mineralization.^63^ The picrosirius analysis in this study demonstrated an increase in the amount of collagen type I in both experimental groups and for collagen type III in BG/SPG. It is well-known that collagen type I plays an important role in structural and mechanical support and collagen type III is important during healing processes,^64,65^ being present in considerable amounts in bone fracture callus, being replaced later by collagen type I during the remodeling phase.^66^ In this context, collagen type III is responsible for trabecular bone quantity and osteoblastogenesis regulation.^67^ Miedel et al. observed that the decrease of collagen type III amount in a tibial bone defect in mice impaired bone formation and remodeling during the healing process.^66^ Taken together, the results of the present work suggest that the positive results of BS/SPG on the increase of collagen type III synthesis at the area of the defect suggests that the composite may promote fibroblast activity and collagen synthesis, indirectly contributing to the extracellular matrix organization during bone healing. This result may have contributed to the acceleration of the process of bone healing, corroborating the findings of the histomorphometry.

Immunohistochemical evaluation revealed the expression of both RUNX-2 and OPG across all analyzed groups. RUNX-2 is a crucial transcription factor involved in the early stages of osteoblast development, primarily responsible for guiding mesenchymal progenitor cells toward osteogenic differentiation. Its expression is linked to the activation of several key osteoblastic genes, including osterix, osteocalcin, and alkaline phosphatase.^68,69^ This regulatory role may explain the enhanced differentiation of osteoblasts and the subsequent formation of bone tissue observed in the treated groups.^68,70^ OPG, also known as osteoclastogenesis inhibitory factor (OFIF) or a member of the tumor necrosis factor receptor superfamily 11b (TNFRSF11B), is a cytokine receptor encoded by the TNFRSF11B gene.^71,72^ *t* is widely produced not only by bone-related cells but also by epithelial tissues of the gastrointestinal and respiratory tracts, skin, vascular endothelial cells, and components of the immune system such as B-cells and dendritic cells.^73,74^ In this context, the enhanced staining of both RUNX-2 and OPG in the BG and SPG-treated groups suggests a favorable influence of these biomaterials on osteogenic and regulatory signaling, supporting their role in the bone repair process.

Taken together, these findings position BG/SPG scaffolds as a promising and biologically safe strategy for promoting bone regeneration in osteoporotic defects, offering a clinically relevant alternative for improving outcomes in patients with compromised bone healing capacity.

## Conclusions

This study demonstrated that BG/SPG scaffolds present significant therapeutic potential for applications in bone tissue engineering, particularly under osteoporotic conditions. Morphological and chemical characterizations confirmed the successful synthesis and structural integrity of both components: BG exhibited irregular, dense particles, while SPG displayed a fibrillar collagen-like structure. FTIR analyses supported the presence of silicon, phosphate, amine, and carbonyl groups, validating the expected composition of both materials. *In vitro* assays using MC3T3-E1 cells showed that BG/SPG scaffolds promoted greater mineralization than BG alone, indicating enhanced osteogenic potential.

*In vivo* evaluations further reinforced these findings. Histological analysis showed no signs of inflammation or material rejection, confirming the biocompatibility of both scaffolds. Histomorphometric analysis revealed increased bone volume and a higher number of osteoblasts at the defect site in animals treated with BG/SPG scaffolds. Picrosirius staining indicated a significant increase in type III collagen deposition, which plays a key role in early phases of bone repair and remodeling. Immunohistochemical analysis demonstrated the expression of RUNX-2 and OPG, markers associated with osteoblast differentiation and regulation of bone turnover, supporting the biological activity of the scaffolds. In addition, the micronucleus assay confirmed that neither BG nor BG/SPG induced genotoxic effects under the tested conditions.

Taken together, these results suggest that the incorporation of an organic matrix such as SPG into BG enhances its regenerative performance, offering a safe and bioactive alternative for the treatment of bone defects. While these findings are encouraging, further studies are needed to evaluate the long-term behavior and therapeutic impact of BG/SPG scaffolds in critical-size bone defects and other pathological conditions.

## Abbreviations

BG: Bioglass 45S5
SPG: spongin-like collagen
FTIR: Fourier-transform infrared spectroscopy
BV/TV: higher bone volume
Ob.S/BS: osteoblast surface
N.Ob/T.Ar: osteoblast number
HCA: hydroxycarbonate apatite
BS: biosilica
Na_2_HPO_4_: sodium bibasic phosphate
MC3T3-E1: rat calvarial preosteoblast cells
CHOK-1: Chinese hamster ovary
MMS: methylmethanesulfonate
NDI: nuclear division index
CNM: mineralization negative control
CPM: mineralization positive control
βGP: β-glycerophosphate
OVX: ovariectomy
EDTA: ethylenediaminetetraacetic acid
Vv: collagen volume fraction
Pt: points
PBS: phosphate-buffered saline
RUNX-2: anti-Runt-related transcription factor 2
OPG: anti-Osteoprotegerin
ANOVA: analysis of variance
OFIC: osteoclastogenesis inhibitor factor
σ: compressive strength
*E*: elastic modulus
